# Science tikkun: A framework embracing the right of access to innovation and translational medicine on a global scale

**DOI:** 10.1371/journal.pntd.0007117

**Published:** 2019-06-06

**Authors:** Peter J. Hotez

**Affiliations:** 1 Texas Children’s Hospital Center for Vaccine Development, Departments of Pediatrics and Molecular Virology and Microbiology, Baylor College of Medicine, Houston, Texas, United States of America; 2 Center for Medical Ethics and Health Policy, Baylor College of Medicine, Houston, Texas, United States of America; 3 Department of Biology, Baylor University, Waco, Texas, United States of America; 4 James A. Baker III Institute for Public Policy, Rice University, Houston, Texas, United States of America; 5 Scowcroft Institute of International Affairs, Bush School of Government and Public Service, Texas A&M University, College Station, Texas, United States of America; Instituto de Ciências Biológicas, Universidade Federal de Minas Gerais, BRAZIL

We’re entering an era when global health is being redefined because of the great progress in vaccination and mass drug administration programs on the one hand, yet on the other hand, there is a changing landscape of social determinants, including urbanization, human migrations, rising antiscience, and a paradigm shift in poverty and poverty-related neglected diseases, known as blue marble health. Science tikkun offers a framework for ensuring that the world’s poor continue to receive access to innovation and technologies in this new world order.

In the almost 2 decades since the start of the Millennium Development Goals (MDGs), later transitioning to the Global Goals for Sustainable Development, we have seen dramatic public health gains in terms of the global reductions in the world’s poverty-related neglected diseases. Two of the most dramatic improvements have been in terms of deaths from childhood-preventable vaccines and disability from the neglected tropical diseases (NTDs). Regarding the former, the Global Burden of Disease (GBD) Study reports a 40–75% reduction in deaths of children under the age of five between the years 2000 and 2015 [1], mostly due to expanded vaccine coverage and introduction of the rotavirus and pneumococcal vaccines—activities led by Gavi, the Vaccine Alliance [2]. For NTDs, we have seen almost (but not quite) as dramatic decreases in the disability-adjusted life years (DALYs) from the seven major diseases targeted by “rapid impact” packages of donated medicines that have now reached more than 1 billion people [3, 4].

Although these gains are impressive, there is still a lot of global health work to be done. Indeed, many of our gains in vaccines and NTDs are under threat from a new group of social determinants and forces that could undermine or even reverse progress made since 2000. For example, because of antivaccine activities and lobbying groups that gained ascendancy more or less contemporaneously with the MDGs, we are seeing thousands of measles cases and deaths return to Europe, and now many counties in the American West have large numbers of unvaccinated children vulnerable to measles and other childhood infections [2, 5]. Children are literally dying as a consequence of an antiscience movement. In Latin America, the political instability and collapse of health systems in Venezuela has also promoted the reemergence of measles cases and deaths there and in neighboring Brazil and Colombia [6].

For NTDs, the gains achieved through integrated mass drug administration are also being undermined by Venezuela’s economic collapse [7], as well as conflict and wars in the Middle East, central Asia, and sub-Saharan Africa [8]. NTDs are also reemerging and rising as a consequence of urbanization [9], population shifts and human migrations [10], climate change [11], and other human-associated activities linked with the modern Anthropocene era [12].

The consequences of two sets of opposing forces—reductions in global disease burdens due to expanded use of vaccines and essential medicines for NTDs versus antiscience movements and Anthropocene forces—have produced an interesting quilt or patchwork of poverty-related neglected diseases. Today, some of the highest rates of these conditions likely occur among the world’s estimated 300–400 million indigenous or aboriginal populations [13]. However, on a larger scale, analyses of data from both the GBD and the World Health Organization (WHO) reveal that most of the world’s neglected diseases and NTDs are actually found among the poor living in the wealthiest economies, especially the group of 20 nations (G20) together with Nigeria, which has an economy greater than the bottom tier of G20 countries [14, 15]. The term “blue marble health” has been used to describe how the “poorest of the rich” are now uniquely vulnerable to disease [16]. NTDs are also paradoxically widespread among the poor in technologically sophisticated countries such as China, India, Iran, and Pakistan, each of these nations with capabilities to produce nuclear weapons [17]. Therefore, the world has profoundly changed in a way that suggests rapid progress in disease control, although vulnerable and impoverished populations living amid great wealth and technical sophistication have been left behind. Such populations remain under constant threat from war, urbanization, population migrations, and climate change.

**Fig 1:**
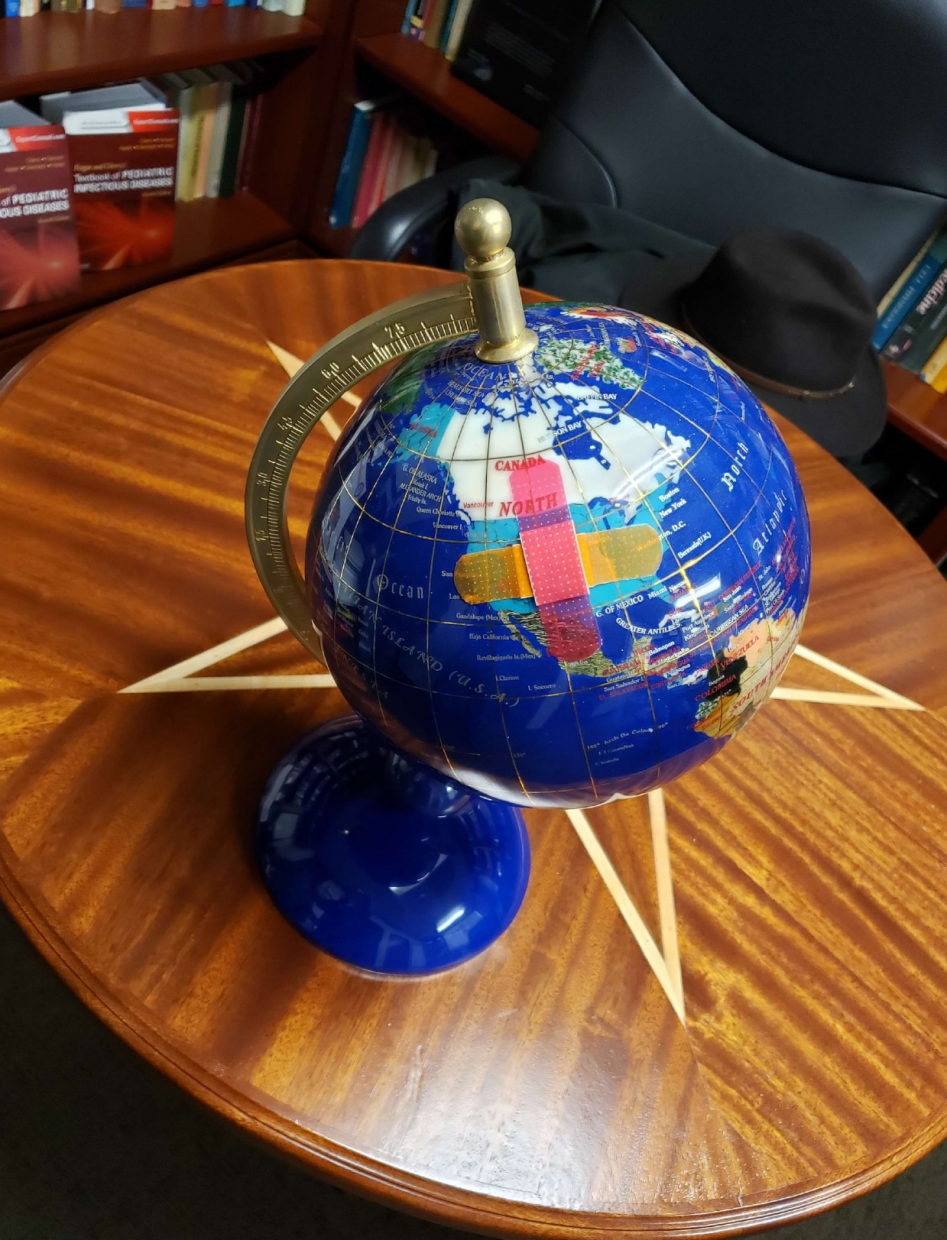
Tikkun olam—Repairing the world.

There is an urgent need to repair the gaps left from these modern 21st century forces. According to some religious scholars, the ancient Jewish framework of repairing the parts of the world still left undone after the creation arose some 500 years earlier during the 16th century (Fig 1) [18]. In his Lurianic Kabbalah, the mystic Rabbi Isaac Luria wrote about reconnecting or repairing the world and cosmos through good works and great deeds [18].

In 2017, I first wrote on the concept of “science tikkun” as a means of “repair and redemption through science” [18]. My original definition focused mostly on science diplomacy and international scientific cooperation, citing the examples of joint United States–Soviet cooperation to develop and deploy vaccines for smallpox and polio for purposes of disease eradication [18–20]. Science tikkun also embraces programs of public engagement by scientists, especially US scientists interacting with the US press, military, and educational sectors [18].

**Fig 2:**
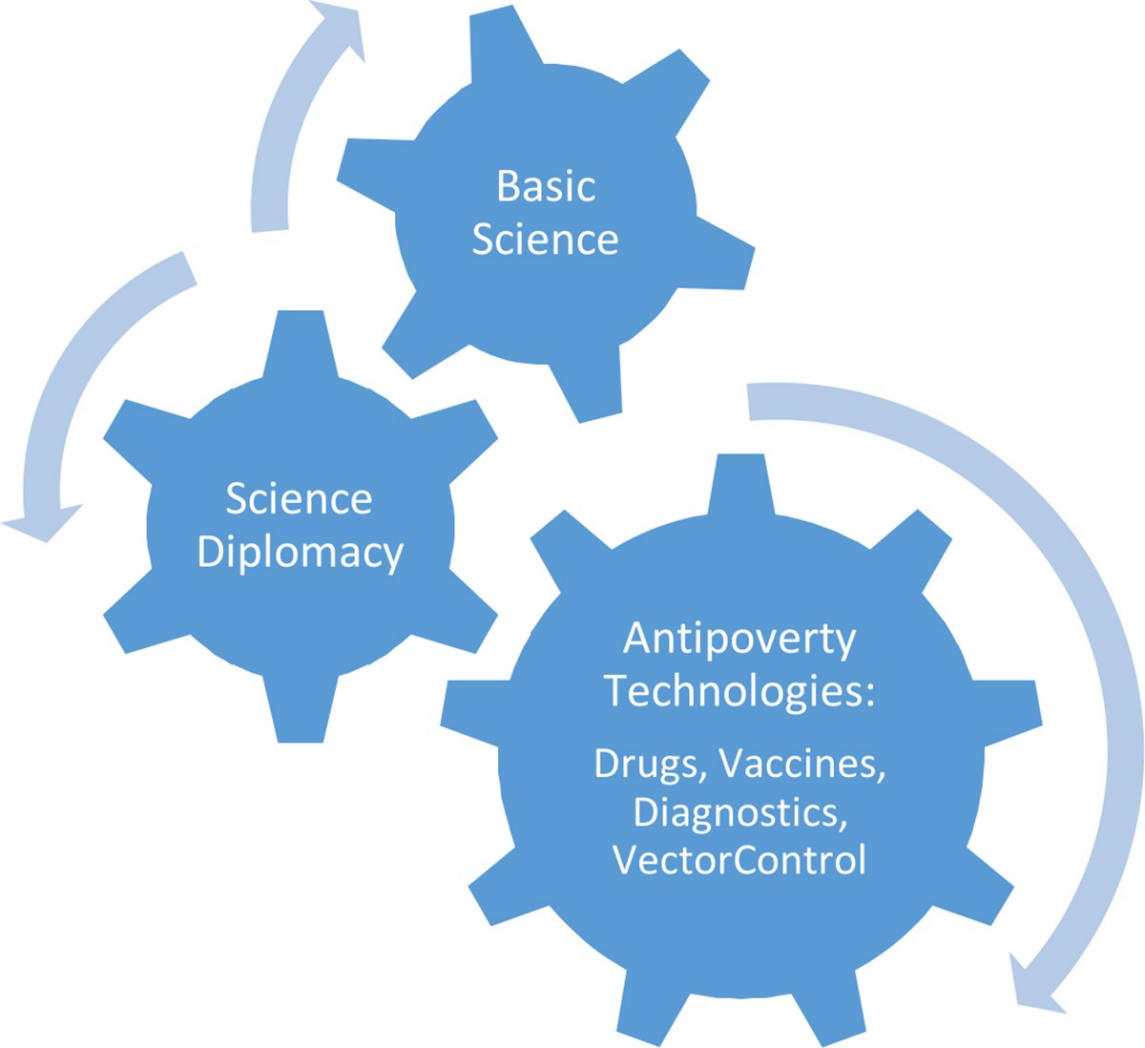
The pillars of science tikkun.

The new world order of science and technology gaps engendered from the opposing forces of successes due to global vaccine and NTD programs versus opposing social determinants of shifting poverty and blue marble health, urbanization, war and conflict, and antiscience movements affords us an opportunity to expand our science tikkun definitions. Here, I redefine it as initiatives led by scientists to address the innovation gaps in global health and neglected diseases allowing illness and disease not only among the world’s vulnerable populations but especially among the huge numbers of poor living amid wealth and prosperity. A fundamental tenet of science tikkun is that vulnerable populations have a fundamental right to access innovation [21]. In this context, science tikkun can take on several different dimensions (Box 1 and Fig 2):

**Box 1. The pillars of science tikkun: The right of access to innovation among the global poor**

- Cutting-edge basic science approaches for neglected diseases
- Antipoverty technologies: drugs, vaccines, diagnostics, and vector control
- Science diplomacy and combating the rise of antiscience

First, basic research on the poverty-related neglected diseases would greatly benefit by expanding its footprint into some of the latest developments in the biochemical, physical, and engineering sciences, including gene editing, functional and comparative OMICs, single-cell combinatorial indexing RNA sequencing, and systems biology and immunology, just to name a few approaches [21]. In some cases, resource-poor nations that have invested heavily in nuclear technologies, including India, Iran, and Pakistan, for example, could see important benefits by redirecting their scientific and technical prowess into basic science for the neglected diseases.

Second, science tikkun embraces translational medicine to develop new drugs, vaccines, diagnostics, and vector control approaches for NTDs and other poverty-related neglected diseases. Such tools are sometimes known as “antipoverty” technologies because of the poverty-promoting disabilities resulting from these diseases [22–25]. Today, the development of antipoverty technologies is being led by academic institutions and nonprofit product development partnerships, but increasingly, there are links with product manufacturers in a group of nations sometimes known as innovative developing countries [26] and some of the multinational pharmaceutical companies. In the future, the new Bill & Melinda Gates Medical Research Institute (Gates MRI) may also play an important role in antipoverty translational medicine.

Lastly, science tikkun can address the social determinants that adversely affect access to innovation for the poor, but two areas in particular that stand out are science diplomacy and combating the rise of antiscience. With regard to the former, the original description of science tikkun designated diplomacy as a central tenet, citing the successes of smallpox and polio eradication that were highlighted earlier [18–20]. However, because the rise of antivaccine and other antiscience movements now threatens the introduction of new technologies in areas where they might be the most needed [2, 25], the current and next generation of scientists embarking on innovation for the poor and vulnerable will be required to address this new threat through public engagement and other mechanisms.

Closing the access to innovation and translational medicine gaps for some of the world’s most disenfranchised peoples—aboriginal populations and the poor living amid wealth—remains one of the great science and technology challenges in this relatively new century. Science tikkun offers a potential and overarching framework for these activities.
